# Adipose-Derived Stem Cell Transplantation Attenuates Inflammation and Promotes Liver Regeneration after Ischemia-Reperfusion and Hemihepatectomy in Swine

**DOI:** 10.1155/2019/2489584

**Published:** 2019-11-18

**Authors:** Zhihui Jiao, Yajun Ma, Xiaoning Liu, Yansong Ge, Qianzhen Zhang, Boyang Liu, Hongbin Wang

**Affiliations:** ^1^Department of Veterinary Surgery, College of Veterinary Medicine, Northeast Agricultural University, Harbin 150030, China; ^2^Heilongjiang Key Laboratory for Laboratory Animals and Comparative Medicine, Harbin 150030, China

## Abstract

**Aim:**

To study the anti-inflammatory and liver regenerative effects of adipose-derived mesenchymal stem cells (ADSCs) on a porcine model of ischemia-reperfusion (IR) and hemihepatectomy.

**Methods:**

Eighteen healthy Bama miniature pigs were randomly divided into the sham-operated (sham), untreated IR injury (IRI), and ADSC-transplanted (ADSC) groups. Hepatic IR was established by laparoscopic hemihepatectomy. ADSCs were transplanted directly into the liver parenchyma after the surgery. Hepatic inflammation and liver regeneration were evaluated by histopathological examination and assessment of relevant cytokines and other factors.

**Results:**

ADSC transplantation successfully ameliorated the IRI-induced histopathological damage and the high levels of pro-inflammatory cytokines like IL-1*β*, IL-6, and TNF-*α*. In addition, the ADSCs enhanced the expression of the anti-inflammatory IL-10, regenerative factors including HGF, Cyclin D1, and proliferating cell nuclear antigen (PCNA), and angiogenic factors like VEGF, ANG-1, and ANG-2.

**Conclusions:**

ADSCs attenuated the hepatic IRI-induced inflammatory response and promoted liver regeneration.

## 1. Introduction

The liver is the largest parenchymal organ in mammals, with functions including detoxification, glycogen storage, and synthesis of secretory proteins. The first hepatectomies were performed in 1949 by Ichio Honjo (Kyoto University) [1] and in 1952 by Jean-Louis Lortat-Jacob [2]. Laparoscopic liver resection was first performed in the 1990s [3, 4]. It minimizes damage to the abdominal wall nerves and muscles; reduces blood loss, pain, and postoperative adhesion; and accelerates recovery [5]. However, small-for-size syndrome [6] after large area hepatectomy and partial liver transplantation will lead to postoperative liver failure. The most effective treatment for terminal liver failure is orthotopic liver transplantation (OLT), which however is limited due to organ shortage, immune rejection, high costs, and post-transplantation complications. Therefore, there is an urgent need to devise novel strategies to repair liver damage and promote liver regeneration.

Mesenchymal stem cells (MSCs) inhibit inflammation and immune responses [7], making them a suitable biotherapeutic tool against inflammatory diseases. Studies show that MSCs can differentiate into hepatocyte-like cells, protect against liver damage, reduce liver fibrosis, promote liver repair, and reduce inflammation [8–10]. Adipose-derived mesenchymal stem cells (ADSCs) are a source of adult MSCs and have multipotent differentiation potential [11–13]. Compared to the 2%-20% yield of bone marrow-derived mesenchymal stem cells (BMSCs) from 1 ml bone marrow, one gram of adipose tissue yields 5,000 to 20,000 ADSCs [14]. In addition, adipose tissue is easier to obtain from liposuction surgery compared to the highly invasive bone marrow extraction. ADSCs also showed a therapeutic advantage over BMSCs in animal models of liver injury [15], especially rodent liver injury [16–19]. However, relatively fewer studies have studied the therapeutic effect of MSCs in liver IRI after partial hepatectomy [20, 21].

Rodents and humans differ greatly in terms of their anatomy, physiology, and biochemistry. In contrast, the anatomy and physiology of pigs are comparable to that of humans, and the size of porcine and human organs are similar. Court et al. [22] showed that the segmental anatomy of pig liver can be directly compared to that of the human liver. Therefore, we established an IRI model in miniature pigs using the laparoscopic approach and performed hemihepatectomy [23], in order to analyze the effects of ADSC transplantation on liver regeneration after IRI and surgery.

## 2. Materials and Methods

### 2.1. Animals

Eighteen Bama miniature pigs (weighing 20-25 kg, aged 4-6 months) were procured from the Farm of the College of Life Sciences (Harbin, China) and housed under ambient conditions in the animal facility. Clinical and laboratory tests were conducted to ensure animal health prior to the experiments that were approved by the Animal Care and Use Committee of Northeast Agricultural University (SQ-2018-357).

### 2.2. Isolation and Culture of ADSCs

The adipose tissue was harvested from the abdomen of miniature pigs, minced, washed, and digested for 50 min with 0.01% collagenase I (Biosharp, China) at 37°C with gentle agitation. The enzyme action was terminated by adding L-DMEM containing 10% FBS. The homogenized tissue was filtered through a 75 *μ*m cell strainer to remove debris and centrifuged at 1200 rpm for 10 min. The precipitated cells were incubated with an erythrocyte-lysing reagent (Solarbio, China) for 5 min and washed twice with DMEM (HyClone, USA). The ADSCs were seeded into 25cm^2^ cell culture flasks (Corning, USA) and cultured with L-DMEM supplemented with 10% FBS (Clark, USA), 2 mM L-glutamine, 1 *μ*g/ml penicillin, and 100 *μ*g/ml streptomycin (all from Solarbio, China) at 37°C under 5% CO_2_ (Galaxy 170 S, Eppendorf, Germany). The media were replaced every 2–3 days. The cells were harvested with 0.25% trypsin-EDTA (Beyotime, China) once they were 70%-80% confluent, centrifuged, resuspended in the culture medium, and reseeded in 75cm^2^ cell culture flasks. Cells from passages 3-5 were used for subsequent experiments.

### 2.3. Characterization of ADSCs

The mesenchymal lineage differentiation of the ADSCs into adipogenic, osteogenic, and hepatic cells was evaluated using the respective differentiation media (Cyagen Biosciences, USA). Adipogenic cells were labeled by staining the lipid droplets with 0.5% Oil Red O, osteogenic cells by staining the mineralized matrix with 0.1 mg/ml Alizarin Red (Solarbio, China), and hepatic cells by staining glycogen with PAS solution (Solarbio, China) according to the manufacturers' instructions. Furthermore, ADSCs were incubated with anti-porcine FITC-CD29, FITC-CD34, FITC-CD44, and FITC-CD105 (Abcam, USA) for 60 min, washed twice with PBS, and acquired via flow cytometry. The labeled population was analyzed with FACSD software (BD, USA).

### 2.4. Surgical Procedure and Administration of ADSCs

**Figure 1:**
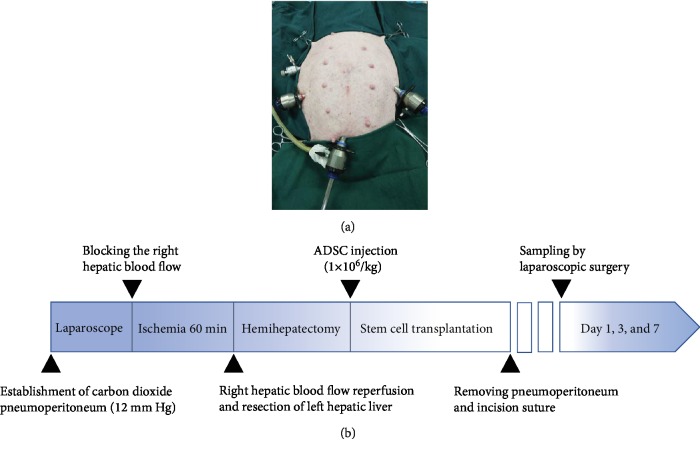
Surgical procedure in miniature pig model. (a) 4-portal approach laparoscopic surgery. (b) Timeline of IRI, hemihepatectomy, and follow-up.

After a 12 h fasting period, all pigs were anaesthetized using 2.5%-4% isoflurane in oxygen with constant monitoring using an invigilator (MP30, Philips, Netherlands), followed by randomization into the sham-operated, untreated IRI model, and ADSC-treated IRI model groups (*n* = 6 each). The animals were laid supine on an operating table maintained at 37°C, and a 4-portal laparoscopy was established (Figure 1(a)). In the sham-operated group, the liver lobe was only flipped and a pneumoperitoneum was established. The IRI model was established by left hemihepatectomy following right hepatic ischemia for 60 min as previously described [24]. Since intravenous injection of MSCs has not been effective in clinically relevant models of IRI and resection [25], the ADSCs (1 × 10^6^ cells/kg) were injected directly into the liver parenchyma immediately after hemihepatectomy in the ADSC groups. The analgesic Tolfedine 4% (Vetoquinol S.A., France) was injected after the operation. The surgical procedure is shown in Figure 1(b). The operated animals were monitored for 7 days, and their behavior, exercise habits, feeding, wound healing, and bowel movements were recorded.

### 2.5. Histological Analysis

The liver tissues were fixed in 4% paraformaldehyde and processed for histological analysis using standard protocols. Hepatic IRI was scored according to the Suzuki classification [26], which considers sinusoidal congestion, vacuolization of hepatocyte cytoplasm, and parenchymal necrosis. Sinusoidal congestion and vacuolization were, respectively, scored as 0: none, 1: minimal, 2: mild, 3: moderate, and 4: severe, and necrosis as 0: none, 1: single cell, 2: 30%, 3: 60%, and 4: >60%.

### 2.6. Peripheral Blood Sample Analysis

Blood samples were collected at different time points (preoperative and postoperative days 1, 3, and 7), and the white blood cells (WBC), neutrophils (NE), and lymphocytes (LY) were measured using a blood routine analyzer (MEK-7222 K, Nihon Kohden, Tokyo, Japan).

### 2.7. ELISA

The serum levels of C-reactive protein (CRP, CK-E50055), vascular endothelial growth factor (VEGF, CK-E95062), angiopoietin-1 (ANG-1, CK-E50049), angiopoietin-2 (ANG-2, CK-E50047), and hyaluronic acid (HA, CK- E50088) were measured using specific ELISA Kits (Suzhou Calvin Biotechnology Co., Suzhou, China) according to the manufacturers' instructions.

### 2.8. Real-Time Reverse Transcriptase-Polymerase Chain Reaction (RT-PCR)

Total RNA was extracted from the liver tissue using Trizol reagent (Invitrogen, China) and assessed by NanoDrop™ One/One (Thermo Fisher Scientific, USA). The RNA was reverse transcribed into cDNA using the PrimeScript™ RT Reagent Kit (Takara, Japan) with gene-specific primers (sequences listed in Table 1). The reaction mix for RT-PCR consisted of 2 *μ*l cDNA, 0.8 *μ*l each of the forward and reverse primers, 6.4 *μ*l H_2_O, and 10 *μ*l SYBR Premix Ex Taq II (Takara, Japan). qRT-PCR was performed in a Light Cycler 480 Real-Time PCR System (Roche Applied Science, Penzberg, Germany) with IL-1*β*, IL-6, IL-10, TNF-*α*, HGF, TGF-*β*, Cyclin D1, ALB, HNF-4a, and *β*-actin primers, and the relative gene expression was calculated by the 2^−*ΔΔ*Ct^ method [27] with *β*-actin as the reference gene. The experiment was performed in triplicates.

### 2.9. Immunohistochemistry (IHC)

Hepatocyte proliferation was analyzed by immunostaining with antiproliferating cell nuclear antigen (PCNA). The liver tissues were fixed in 10% formalin for 24 h, cut into 4 *μ*m thick sections, and incubated with EDTA for antigen retrieval. The slides were then washed in PBS and incubated with 3% H_2_O_2_ for 10 min to quench endogenous peroxidases. After blocking with bovine serum albumin (BSA), the sections were incubated overnight with antiproliferating cell nuclear antigen (PCNA) antibody (Abcam, Cambridge, UK) overnight at 4°C, followed by HRP-conjugated goat anti-rabbit secondary antibody (Beijing ZSGB-Biotechnology Co., Ltd., Beijing, China) at room temperature for 30 min. Color was developed using DAB, and after counterstaining with hematoxylin, the sections were observed under a microscope and immunostaining was quantified with IPP 6.0 software (Media Cybernetics, USA).

### 2.10. Statistical Analysis

All data are expressed as means ± SD and analyzed using SPSS 22.0 software. The groups were compared by one-way ANOVA and *P* < 0.05 was considered statistically significant.

## 3. Results

### 3.1. Isolation and Characterization of ADSCs

**Figure 2:**
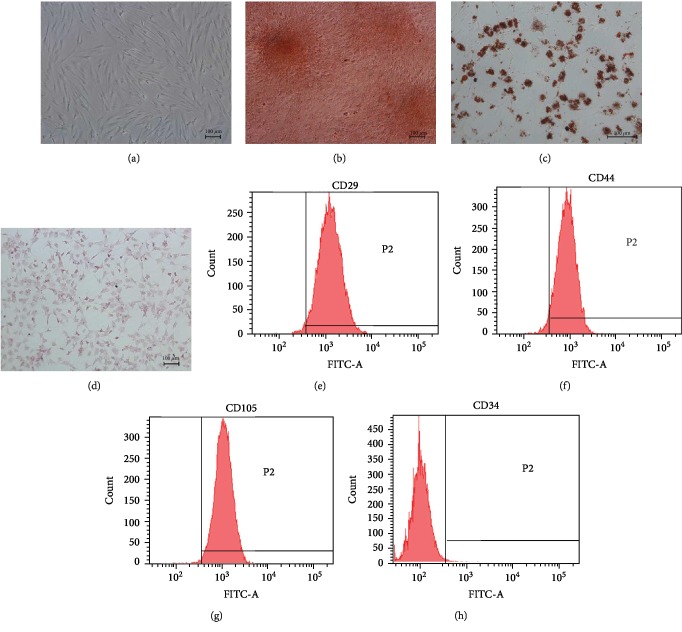
Identification and characterization of ADSCs. (a–d) Representative images of (a) passage three spindle-shaped ADSCs (magnification 100x), (b) Alizarin Red-stained calcium nodules (magnification 100x), (c) Oil Red O-stained lipid droplets (magnification 200x), and (d) PAS-stained glycogen granules (magnification 100x). (e–h) ADSC flow cytometry plots showing percentage of (e) CD29, (f) CD44, and (g) CD105 positive expressions and (h) negative CD34 expression.

ADSCs isolated from the porcine adipose tissue adhered to the plastic dishes within 24 h of culture and exhibited the typical spindle shape after 2-3 days (Figure 2(a)). The differentiation potential of the ADSCs into the osteogenic, adipogenic, and hepatic lineages were assessed by established assays. Alizarin Red staining showed presence of calcium crystals (Figure 2(b)), Oil Red O staining showed lipid droplets (Figure 2(c)), and PAS staining showed glycogen deposits (Figure 2(d)) in 80% of the cells cultured in the osteogenic, adipogenic, and hepatic differentiation media for 21, 14, and 21 days, respectively. Finally, the ADSCs were positive for CD29 (98.6%), CD44 (94.5%), and CD105 (99.2%) and negative for CD34 (0.7%), thus confirming the characteristic immunophenotype (Figures 2(e)–2(h)). Taken together, multipotent ADSCs were successfully enriched from porcine adipose tissue.

### 3.2. ADSCs Alleviated the Histopathological Damage Caused by IRI

**Figure 3:**
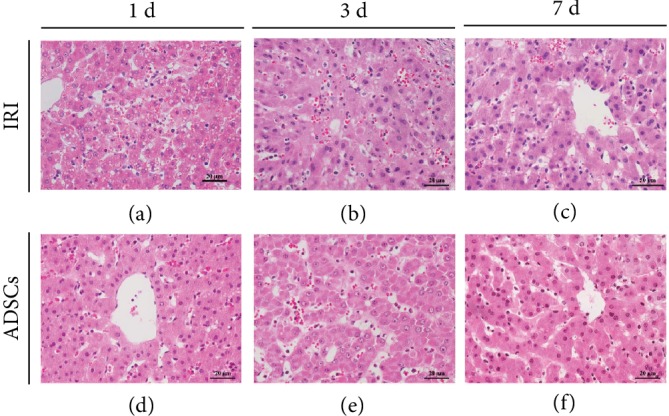
Histopathological changes in the liver after IRI and effect of ADSCs. Representative HE-stained images showing the liver parenchyma in the (a–c) untreated IRI and (d–f) ADSC-transplanted group at day 1, 3, and 7 (original magnification 400x).

The pathological changes in the liver of the differentially treated miniature pigs were assessed by hematoxylin and eosin staining. As shown in Figure 3, the liver of the sham-operated animals had a normal lobular structure, with clear liver cord and no signs of damage. Ischemia-reperfusion and hemihepatectomy injury destroyed the hepatic cell cord structure, resulting in hepatic sinus congestion, enucleation of hepatocytes, homogenized cytoplasm, necrotic foci, and hepatocyte vacuolization. Furthermore, massive inflammatory cell influx and exudation was seen in the hepatic parenchyma on day 3 and 7 post-operation. Local transplantation of ADSCs decreased necrosis and vacuolar degeneration within 24 h, while the hepatocytes were swollen. After 3 days of transplantation, hepatic sinus congestion and inflammatory cells were still visible, but significantly reduced by the 7th day, and the hepatocyte cord structure was also repaired. Morphological examination of liver tissue showed that the degree of hepatic sinus congestion, liver tissue necrosis, and vacuolar degeneration was significantly lower in the ADSC group than in the IRI group, although there was no significant difference between the two groups (Table 2). Taken together, transplantation of ADSCs can reduce hepatic degeneration, inflammatory cell infiltration, and liver cell damage.

### 3.3. ADSCs Decreased the Inflammatory Response Induced by IRI

**Figure 4:**
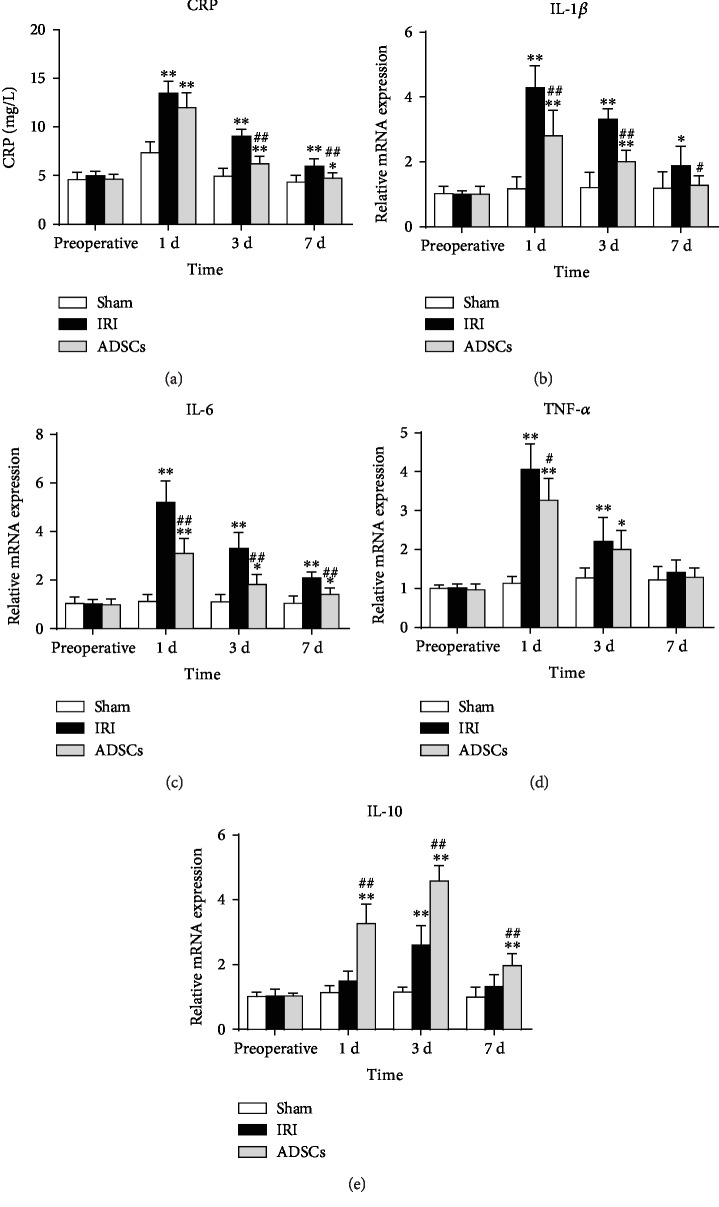
Effects of ADSCs on the serum indices of inflammatory response. Serum levels of (a) CRP; (b–d) IL-1*β*, IL-6, and TNF-*α*; and (e) IL-10 in the different groups. CRP: C-reactive protein; IL-1*β*: interleukin-1 beta; IL-6: interleukin-6; TNF-*α*: tumor necrosis factor alpha; IL-10: interleukin-10; IRI: ischemia-reperfusion injury; ADSCs: adipose-derived mesenchymal stem cells. The data were expressed as mean ± SD. ^∗^*P* < 0.05, ^∗∗^*P* < 0.01, versus the sham group. ^#^*P* < 0.05, ^##^*P* < 0.01, versus the IRI group.

IRI significantly increased WBC, LY, and NE counts in the peripheral blood 1 day after reperfusion (*P* < 0.01). ADSC transplantation significantly lowered the day 1 post-operative WBC count compared to the untreated IRI group (*P* < 0.05), as well as day 1 and day 3 post-operative LY and NE counts (*P* < 0.01) (Table 3). Thus, ADSC transplantation decreased immune cell circulation, which might be instrumental in alleviating post-ischemia liver injury and the inflammatory reactions. To further validate this hypothesis, we analyzed the levels of inflammation markers including C-reactive protein (CRP) and various cytokines. As showed in Figure 4, the serum CRP levels peaked 1 day after surgery in both the untreated IRI and ADSC groups and was significantly higher compared to the sham-operated group (*P* < 0.01). By day 3, however, the CRP levels were restored in the ADSC-transplanted animals (*P* < 0.01 compared to IRI), while they remained high in the untreated IRI group (*P* < 0.01 compared to the sham-operated group). In addition, the pro-inflammatory TNF-*α*, IL-1*β*, IL-6, and IL-10 mRNAs were significantly increased in the IRI group compared to the sham-operated group (*P* < 0.01), and that of IL-1*β*, IL-6, and TNF-*α* were reduced by ADSC transplantation within 1 day after surgery (*P* < 0.01, *P* < 0.05 for TNF-*α*). The anti-inflammatory cytokine IL-10 was also elevated after IRI and increased further after ADSC transplantation compared to the untreated IRI pigs (*P* < 0.01). Taken together, ADSCs relieved the inflammatory response induced by IRI.

### 3.4. ADSCs Promote Liver Regeneration after IRI

**Figure 5:**
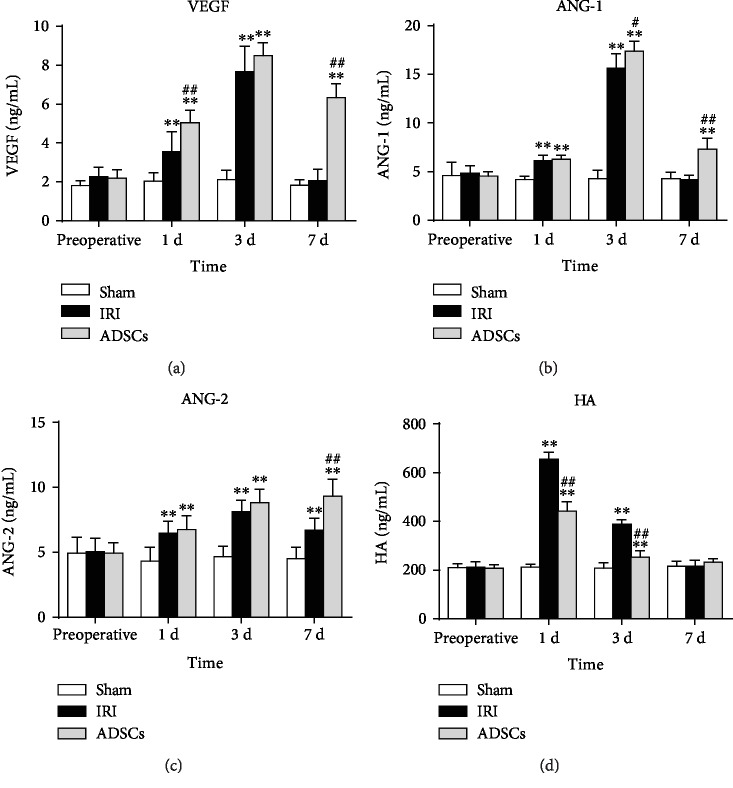
Effects of ADSCs on serum levels of angiogenic factors. Serum levels of (a) VEGF, (b) ANG-1, (c) ANG-2, and (d) HA in the different groups. VEGF: vascular endothelial growth factor; ANG: angiopoietin; HA: hyaluronic acid; IRI: ischemia-reperfusion injury; ADSCs: adipose-derived mesenchymal stem cells. The data were expressed as mean ± SD. ^∗^*P* < 0.05, ^∗∗^*P* < 0.01, versus the sham group. ^##^*P* < 0.01, versus the IRI group.

Liver regeneration following any injury is driven by hepatocyte proliferation and neovascularization of the hepatic parenchyma. Therefore, we also analyzed the levels of various proangiogenic and regenerative factors following ADSC transplantation in the IRI model. The serum levels of angiogenic factors including VEGF, ANG-1, and ANG-2 increased gradually following IRI and peaked on day 3 and declined thereafter (Figure 5). While VEGF and ANG-1 levels were restored to normal levels 7 days after hemihepatectomy, that of ANG-2 was still significantly higher compared to the sham-operated group on day 7 (*P* < 0.01). ADSC transplantation significantly increased the levels of all angiogenic factors compared to the untreated IRI group (*P* < 0.01). In addition, serum HA levels increased significantly on the 1st and 3rd day post-hemihepatectomy and decreased after ADSC transplantation (*P* < 0.01) to normal preoperative levels 7 days after surgery.

**Figure 6:**
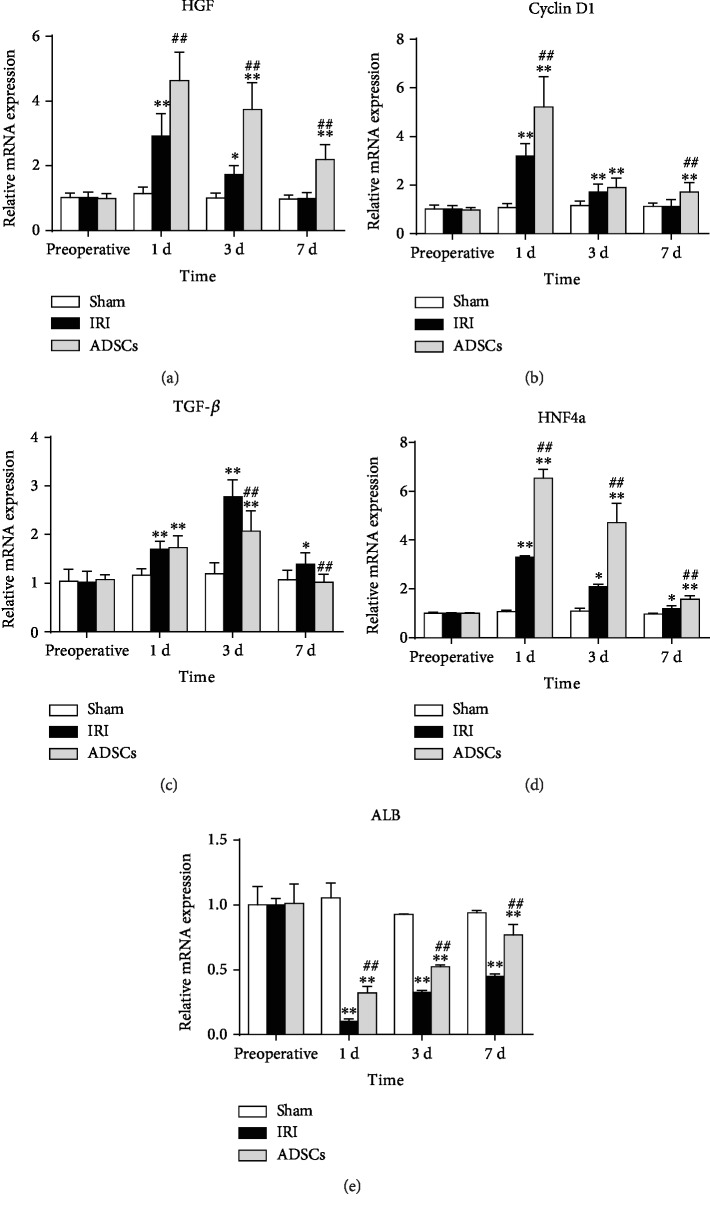
Expression levels of genes related to liver regeneration. Relative mRNA levels of (a) HGF, (b) Cyclin D1, (c) TGF-*β*, (d) HNF-4a, and (e) ALB. HGF: hepatocyte growth factor; TGF-*β*: transforming growth factor-beta; HNF-4a: hepatocyte nuclear factor 4A; ALB: albumin; IRI: ischemia-reperfusion injury; ADSCs: adipose-derived mesenchymal stem cells. The data were expressed as mean ± SD. ^∗^*P* < 0.05, ^∗∗^*P* < 0.01, versus the sham group. ^##^*P* < 0.01, versus the IRI group.

**Figure 7:**
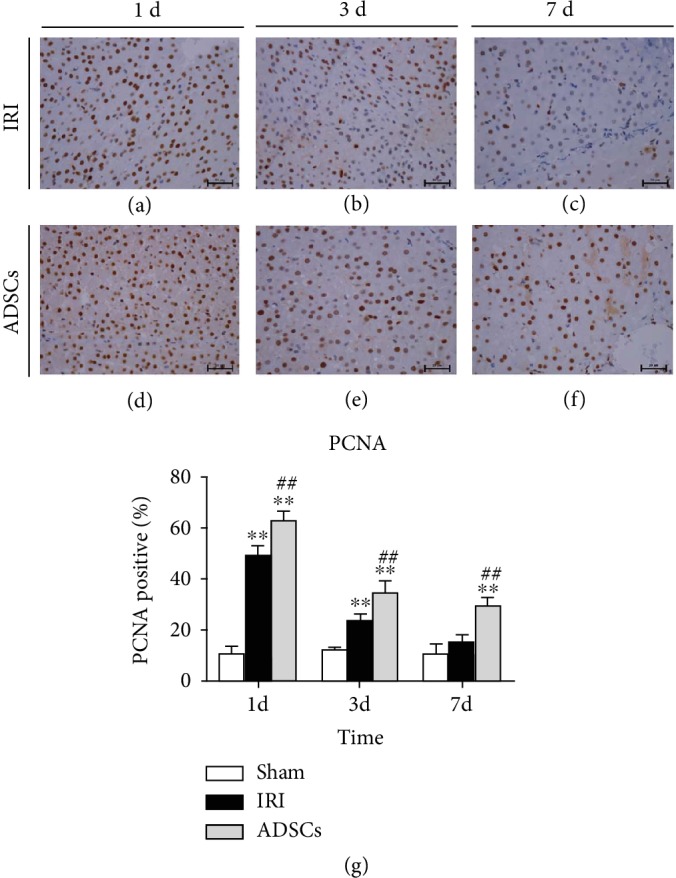
Effect of ADSCs on PCNA expression. Representative IHC images showing *in situ* PCNA expression in the (a–c) IRI and (d–f) ADSC groups at day 1, 3, and 7. (g) Percentage of PCNA-positive cells in the different groups. IRI: ischemia-reperfusion injury; ADSCs: adipose-derived mesenchymal stem cells. ^∗^*P* < 0.05, ^∗∗^*P* < 0.01, versus the sham group. ^#^*P* < 0.05, ^##^*P* < 0.01, versus the IRI group.

The hepatocyte growth factor (HGF) and Cyclin D1 levels increased significantly on the first day after surgery and started declining from the third day onwards to preoperative levels within one week (Figure 6). ADSC transplantation significantly increased both HGF (*P* < 0.01) and Cyclin D1 (*P* < 0.01) levels one day post-surgery. On the other hand, TGF-*β* mRNA levels were significantly elevated in the IRI and ADSC groups and peaked on day 3, although the levels were significantly lower in the latter on day 3 and 7 compared to the IRI group (*P* < 0.01). After hemihepatectomy, although albumin (ALB) expression decreased sharply in both groups, stem cell transplantation significantly increased ALB mRNA expression in liver parenchyma at day 1, 3, and 7, compared with the model group (*P* < 0.01). On the 7th day after operation, the ALB level in the ADSC group was closer to that in the sham-operated group. For the expression of the hepatic-specific gene hepatocyte nuclear factor 4A (HNF-4a), all stem cell-transplanted animals showed a significantly higher expression rate of HNF-4a at all time points after the operation (*P* < 0.01), compared with IRI group animals. Finally, the number of PCNA-positive cells in the liver parenchyma increased significantly on days 1 and 3 after surgery (Figure 7) and peaked on the first postoperative day in the ADSC-transplanted group. High PCNA expression was maintained in the latter on days 1, 3, and 7 post-surgery (*P* < 0.01 compared to IRI, Figure 7(g)), while it was restored to preoperative levels in the untreated IRI group by day 7.

## 4. Discussion

We hypothesized that adipose-derived mesenchymal stem cells (ADSCs) can protect the liver after ischemia-reperfusion- (IR-) induced damage. As proof of concept, we established a partial hepatectomy and IRI model by laparoscopic surgery in Bama miniature pigs and treated them with ADSC transplantation. In a previous study, we showed that ADSCs alleviated hepatocyte apoptosis induced by liver I/R and hepatectomy [28], enhanced the activity of antioxidant enzymes by alleviating lipid peroxidation, and inhibited autophagy [29]. In this study, we found that ADSCs alleviated IRI-induced liver damage by attenuating the inflammatory response and promoting liver regeneration.

The pathological basis of liver IRI is inflammation, which is manifested by hepatocyte degeneration and necrosis, massive leukocyte infiltration, and microvascular thrombosis. These pathological changes were also observed in our model, along with increased numbers of inflammatory cells in the peripheral blood. Ischemia triggers an increase in the number of circulating WBCs and NE infiltration in the reperfused tissue. The LY promote NE influx into the ischemic microenvironment by secreting IL-17 [30], and the NEs in turn activate the endothelial cells, and attract more parenchymal cells and monocytes to the lesion. These cells release inflammatory mediators into the bloodstream and activate the systemic complement system [31]. Seki reported that ADSCs reduced fibrosis and inflammatory cell infiltration in the liver of mice with cirrhosis [32]. Consistent with previous studies, the IRI-induced surge in inflammatory cells seen in our model was alleviated by ADSC transplantation, which also reduced hepatic degeneration, inflammatory cell infiltration, and hepatocyte damage and improved liver histopathological changes. Similar findings have been reported by other research groups [33, 34].

Studies show that hepatic IRI triggers a “waterfall effect” of IL-1*β*, IL-6, TNF-*α*, and other inflammatory cytokines, resulting in a systemic inflammatory immune response. Kupffer cells, the resident macrophages in the liver, are activated during blood perfusion and produce inflammatory cytokines that aggravate liver IRI. The Kupffer cell-derived TNF-*α* and IL-1*β* activate the endothelial cells and hepatocytes, resulting in the production of large amounts of ROS, increased expression of adhesion molecules, and microcirculatory disorders. IL-6 is produced by the Kupffer cells in the later stages of IRI [35] and induces fever, cerebral corticosteroid release, and other acute phase reactions along with TNF-*α* and IL-1, resulting in an uncontrolled inflammatory response [36]. In this study, hepatic levels of TNF-*α*, IL-1*β*, and IL-6 spiked within a day of hemihepatectomy and were attenuated after ADSC transplantation. A similar finding was reported for ventilator-induced lung injury in rats [37], and Li et al. showed that ADSCs reduced lymphocyte infiltration in a rabbit model of autoimmune dacryoadenitis and enhanced the IL-10 levels [38]. Consistent with this, the IL-10 levels were also elevated in our model of hepatic IRI after stem cell transplantation. The post-IRI increase in IL-6 secretion by Kupffer cells promotes CRP synthesis in hepatocytes, which further promotes an inflammatory response [39]. A previous study showed that ADSC transplantation significantly reduced serum CRP levels in type 2 diabetic rats [39]. In agreement with the above, we found that ADSCs reduced CRP synthesis after hepatic IRI. Taken together, ADSCs can alleviate the IRI-induced inflammatory response by inhibiting production of proinflammatory factors and increasing IL-10 levels.

Endothelial cells are the most abundant nonparenchymal cells in the liver and the main site of HA metabolism. High serum HA content is therefore an indicator of the degree of endothelial cell damage [40]. In our study, HA levels were significantly reduced after ADSC transplantation, indicating that these cells can restore damaged endothelial cells. Liao et al. showed that ADSCs protected against liver fibrosis by preventing HA accumulation and thus protecting endothelial cells [39]. Endothelial cells line the inner walls of the blood vessels and are involved in phagocytosis, antigen presentation, blood flow regulation, and secretion of cytokines like VEGF and TGF-*β* [41, 42]. Studies show a dual protective effect of VEGF on IRI, especially in hepatic tissues. VEGF levels increase early during reperfusion, which increases vascular permeability, promotes inflammatory cell infiltration, and aggravates inflammatory responses [43]. It is also a mitogen that accelerates endothelial cell regeneration and neovascularization, thus allowing the organs to adapt to ischemia and hypoxia. Studies show that ADSC transplantation upregulates VEGF and enhances liver regeneration in rats with IRI and hepatic resection by CCl_4_-induced hepatic fibrosis [14]. ANG-1 and ANG-2 also play vital roles in angiogenesis, and ADSC transplantation in rats with femoral artery injury increased the ANG-1 levels [44]. In addition, Watt used MSCs to treat spinal cord injury and reported increased secretion of VEGF and ANG-2 in the spinal cord [45]. Consistent with these previous findings, ADSCs increased the secretion of VEGF, ANG-1, and ANG-2 after hepatic IRI.

Liver regeneration involves both parenchymal and nonparenchymal cells [46–48]. The former includes mature differentiated liver cells that account for 70% to 80% of the liver parenchyma, and the latter include Kupffer cells, endothelial cells, and stellate cells. In the resting state, the mature liver cells exist in a highly differentiated state, but can rapidly proliferate following injury in order to repair the damaged tissue. The nonparenchymal cells are also activated in the damaged liver and secrete regenerative cytokines and growth factors [46, 48], including HGF which initiates liver regeneration [49] by stimulating DNA synthesis and hepatocyte proliferation through the c-Met receptor [50]. Cyclin D1 promotes the G1 to S phase transition and is also a critical factor promoting hepatocyte proliferation [51]. TGF-*β*, a negative regulator of liver regeneration and proliferation, is also activated post-injury to prevent unrestricted growth during liver regeneration. Okamoto et al. found that liver IRI reduced HGF expression, while exogenous HGF promoted liver regeneration [52]. Fouraschen et al. showed that hepatic Cyclin D1 mRNA levels were higher in the mice transplanted with MSCs, while that of TGF-*β* was lower, indicating that MSCs promoted post-injury liver regeneration [53]. Another study showed that TGF-*β* mRNA levels increased in the residual liver tissue 4 h after partial hepatectomy and peaked 72 h after surgery, resulting in DNA synthesis inhibition [46]. In the present study, the HGF and cyclin D1 levels were significantly higher in the ADSC group compared to the untreated IRI group, and the initial spike in TGF-*β* levels following the surgery subsided in the presence of ADSCs. We also studied the effect of ADSC transplantation on the expression of PCNA in hepatocytes. PCNA is a cofactor of DNA polymerase and a reliable indicator of DNA synthesis and cellular proliferation. After ischemia-reperfusion and hemihepatectomy, PCNA expression in the liver tissue of IRI group pigs was upregulated compared with the sham-operated group. Animals treated with ADSC transplantation had significantly higher number of PCNA-positive cells compared to the untreated IRI group on days 1, 3, and 7. That is, ADSCs were still playing a role in promoting liver regeneration on the 7th day after surgery. This is consistent with the studies of Saidi et al. [54] and Ezquer et al. [55] that showed increased liver regeneration after MSC transplantation. It is indicated that adipose-derived mesenchymal stem cells can upregulate PCNA expression at the gene level and promote hepatocyte regeneration.

We also studied the expression of liver-specific genes in regenerative liver parenchyma. Hepatocyte nuclear factor (HNF) is an important component of liver-enriched transcription factors (LETFs) and plays an important role in regulating hepatocyte differentiation and maintaining hepatocyte biological functions [56]. Hepatocyte nuclear factor 4A (HNF-4a) is highly expressed in differentiated and mature hepatocytes and is an important transcription factor regulating hepatocyte differentiation. In this study, stem cell transplantation significantly increased HNF-4a expression after hepatic ischemia-reperfusion injury. This is consistent with other studies [57], demonstrating that HNF-4a expression is significantly upregulated during liver regeneration [58]. Contrary to that, albumin (ALB) decreased expression levels after surgery. It is expressed in mature hepatocytes and reflects the ability of hepatocytes to synthesize proteins, generally consistent with liver injury [59]. After partial hepatectomy, the function of ribosome-bound albumin synthesis in hepatocyte endoplasmic reticulum was impaired. With the recovery of liver regeneration, albumin content gradually increased. Our study found that ALB levels were lower in the ADSC and IRI groups, but the ADSC group significantly increased ALB expression levels in days 1, 3, and 7 compared with the IRI group. As also demonstrated in other studies [19, 59], transplantation of stem cells promotes liver regeneration, which accelerates the recovery of ALB levels. One limitation of this study was the absence of regenerative hepatic weight evaluation. Nevertheless, our findings clearly indicate that ADSC transplantation can accelerate liver regeneration after hepatic IRI combined with hemihepatectomy. A previous study showed that ADSC transplantation significantly ameliorate liver function by reducing serum levels of aspartate aminotransferase (AST), alanine aminotransferase (ALT), total bilirubin (T-BIL), and lactate dehydrogenase (LDH) after hepatic IRI combined with hemihepatectomy [29]. However, the mechanism of stem cells in the liver has not yet been explained. It has been known that it may improve liver function by differentiating into hepatocyte-like cells [60]. In addition, stem cells can affect adjacent cells by secreting growth factors and cytokines in a paracrine manner [15, 61]. Ranganathet et al. [62] proposed the concept of mesenchymal stem cell secretome, emphasizing the importance of secreting-related proteins and cytokines to play a role in tissue regeneration. Therefore, further experiments are needed to explore the mechanism of stem cells for stem cell therapy applications.

## 5. Conclusion

ADSC transplantation alleviates liver damage after IRI and hemihepatectomy by reducing secretion of inflammatory cytokines and promotes liver regeneration by increasing the expression of anti-inflammatory, regenerative, angiogenic, and mitotic factors. Therefore, ADSCs are a promising new strategy that can promote liver regeneration and reduce the inflammatory response after liver ischemia-reperfusion injury.

## Figures and Tables

**Table 1:** Gene-specific primers used in the qPCR.

Gene	Primer sequences(5′-3′)
IL-1*β*	Forward 5′-TCTCCAGCCAGTCTTCATTGT-3′
	Reverse 5′-GCCATCAGCCTCAAATAACAG-3′

IL-6	Forward 5′-AGCAAGGAGGTACTGGCAGA-3′
	Reverse 5′-AAGACCGGTGGTGATTCTCA-3′

IL-10	Forward 5′-GGGAGGATATCAAGGAGCACG-3′
	Reverse 5′-CTTGGAGCTTGCTAAAGGCAC-3′

TNF-*α*	Forward 5′-ACCAGCCAGGAGAGAGACAAG-3′
	Reverse 5′-AGCGTGTGAGAGGGAGAGAGT-3′

HGF	Forward 5′-TGATCAACTCAGACGGCCTA-3′
	Reverse 5′-AGCCCCAGCACATATTTCAG-3′

Cyclin D1	Forward 5′-AAGTGCGTGCAGAAGGAAAT-3′
	Reverse 5′-AGGAAGCGGTCCAGGTAGTT-3′

TGF-*β*	Forward 5′-CCATTCGCGGCCAGATT-3′
	Reverse 5′-GCTCCGGTTCGACACTTTC-3′

ALB	Forward 5′-TCGCCTGAGCCAGAGATTTCCC-3′
	Reverse 5′-CCGCCCTGTCATCTGCACATTC-3′

HNF-4a	Forward 5′-CAAGAGGAACCAGTGCCGCTAC-3′
	Reverse 5′-GCTTGACCTGCGAGTGCTGATC-3′

*β*-Actin	Forward 5′-TCTGGCACCACACCTTCT-3′
	Reverse 5′-TGATCTGGGTCATCTTCTCAC-3′

IL-1*β*: interleukin-1 beta; IL-6: interleukin-6; IL-10: interleukin-10; TNF-*α*: tumor necrosis factor alpha; HGF: hepatocyte growth factor; TGF-*β*: transforming growth factor-beta; ALB: albumin; HNF-4a: hepatocyte nuclear factor 4a.

**Table 2:** Pathological score of liver.

Groups	Congestion			Vacuolization			Necrosis		
	1d	3d	7d	1d	3d	7d	1d	3d	7d
IRI	1.88 ± 0.22	1.63 ± 0.65	0.88 ± 0.54	2.00 ± 0.61	1.75 ± 0.75	1.13 ± 0.74	2.50 ± 0.50	1.50 ± 0.35	1.25 ± 0.25
ADSCs	1.13 ± 0.74	0.88 ± 0.54	0.63 ± 0.41	1.63 ± 0.54	1.25 ± 0.56	0.88 ± 0.54	1.88 ± 0.65	1.38 ± 0.65	0.88 ± 0.74
*P* value	0.143	0.176	0.550	0.458	0.390	0.654	0.235	0.780	0.437

IRI: ischemia-reperfusion injury; ADSCs: adipose-derived mesenchymal stem cells.

**Table 3:** Effects of ADSCs on peripheral blood.

Variables	Time	Sham (*n* = 6)	*P* value	IRI (*n* = 6)	*P* value		ADSCs (*n* = 6)	*P* value		
			*P*_1_		*P*_1_	*P*_2_		*P*_1_	*P*_2_	*P*_3_
White blood cell (×10^9^/L)	Preoperative	19.13 ± 4.32		16.50 ± 3.95			17.82 ± 2.10			
	1d	22.03 ± 3.26	0.183	33.57 ± 10.14^▲▲,∗∗^	**0.000**	**0.006**	23.70 ± 2.50^▲▲,#^	**0.000**	0.654	**0.016**
	3d	19.42 ± 3.91	0.894	27.08 ± 5.93^▲,∗∗^	**0.010**	**0.007**	20.12 ± 2.04^#^	0.078	0.780	0.013
	7d	16.28 ± 2.88	0.190	20.92 ± 3.57^∗^	0.249	**0.013**	18.05 ± 1.88	0.113	0.302	0.103

Neutrophil (×10^9^/L)	Preoperative	7.85 ± 0.94		7.07 ± 1.20			7.10 ± 0.82			
	1d	14.27 ± 1.96^▲▲^	**0.000**	24.25 ± 3.52^▲▲,∗∗^	**0.000**	**0.000**	18.70 ± 2.10^▲▲,∗,##^	**0.000**	**0.010**	**0.002**
	3d	8.53 ± 3.02	0.563	17.23±3.07^∗∗^	0.316	**0.000**	12.22 ± 1.93^▲▲,∗,##^	**0.000**	**0.033**	**0.006**
	7d	9.27 ± 1.55	0.237	9.42 ± 1.21	0.115	0.830	8.58 ± 0.60	0.106	0.334	0.243

Lymphocyte (×10^9^/L)	Preoperative	7.20 ± 2.57		7.17 ± 2.12			7.65 ± 0.39			
	1d	11.48 ± 1.96^▲^	**0.0140.**	18.75 ± 3.4^▲▲,∗∗^	**0.000**	**0.000**	13.00 ± 0.6^▲▲,##^	**0.000**	0.275	**0.001**
	3d	10.87 ± 2.95^▲^	**032**	13.65 ± 1.41^▲▲,∗^	**0.000**	**0.025**	9.57 ± 0.80^▲▲,##^	**0.000**	0.265	**0.002**
	7d	7.17 ± 3.34	0.983	8.40 ± 1.54	0.360	0.335	8.07 ± 0.53	0.242	0.478	0.791

IRI: ischemia-reperfusion injury; ADSCs: adipose-derived mesenchymal stem cells. ^▲^*P*_1_ < 0.05, preoperative comparison with different time points (^▲▲^*P*_1_ < 0.01); ^∗^*P*_2_ < 0.05, compared to the sham group (^∗∗^*P*_2_ < 0.01); ^#^*P*_3_ < 0.05, compared to the IRI group (^##^*P*_3_ < 0.01).

## Data Availability

The data used to support the findings of this study are available from the corresponding author upon request.
